# Flavonoids Extraction from Propolis Attenuates Pathological Cardiac Hypertrophy through PI3K/AKT Signaling Pathway

**DOI:** 10.1155/2016/6281376

**Published:** 2016-04-24

**Authors:** Guang-wei Sun, Zhi-dong Qiu, Wei-nan Wang, Xin Sui, Dian-jun Sui

**Affiliations:** ^1^China-Japan Union Hospital of Jilin University, Changchun 130033, China; ^2^Chinese Traditional Medicine Institute of Ji Lin Province, Changchun 130021, China; ^3^Changchun University of Chinese Medicine, Changchun 130117, China

## Abstract

Propolis, a traditional medicine, has been widely used for a thousand years as an anti-inflammatory and antioxidant drug. The flavonoid fraction is the main active component of propolis, which possesses a wide range of biological activities, including activities related to heart disease. However, the role of the flavonoids extraction from propolis (FP) in heart disease remains unknown. This study shows that FP could attenuate ISO-induced pathological cardiac hypertrophy (PCH) and heart failure in mice. The effect of the two fetal cardiac genes, atrial natriuretic factor (ANF) and *β*-myosin heavy chain (*β*-MHC), on PCH was reversed by FP. Echocardiography analysis revealed cardiac ventricular dilation and contractile dysfunction in ISO-treated mice. This finding is consistent with the increased heart weight and cardiac ANF protein levels, massive replacement fibrosis, and myocardial apoptosis. However, pretreatment of mice with FP could attenuate cardiac dysfunction and hypertrophy* in vivo*. Furthermore, the cardiac protection of FP was suppressed by the pan-PI3K inhibitor wortmannin. FP is a novel cardioprotective agent that can attenuate adverse cardiac dysfunction, hypertrophy, and associated disorder, such as fibrosis. The effects may be closely correlated with PI3K/AKT signaling. FP may be clinically used to inhibit PCH progression and heart failure.

## 1. Introduction

Cardiac hypertrophy is frequently observed in various clinical conditions, including hypertension and aortic stenosis. It can progressively lead to heart failure (HF). HF is the most frequent cause of cardiovascular death worldwide. HF is caused by a pathological state that results in insufficient cardiac output [1–6]. Although there has been great progress in therapy, the mortality and morbidity of HF place it in the top 5 lethal diseases. Currently, the high mortality of HF imposes a significant economic burden in both China and Western countries [7]. Thus, identifying novel preventive and/or therapeutic strategies to counteract this deadly disease is of importance.

Cardiac hypertrophy, which is characterized by increased cell volume and metabolic and biochemical disorders, can also lead to reactivation of fetal cardiac genes such as atrial natriuretic factor (ANF) and *β*-myosin heavy chain (*β*-MHC) [8–10]. Currently, cardiac hypertrophy is attracting attention because it can progressively lead to HF [11–15]. Some specific factors with cardioprotective properties have been identified through the preventative mechanisms of minimizing cardiac hypertrophy. Some cellular/molecular pathways, including the mitochondrial pathway, phosphoinositide 3-kinase (PI3K), AKT, and mammalian target of rapamycin (mTOR), are involved in the progression of cardiac hypertrophy [1, 4, 16].

Propolis, a complex mixture of naturally sticky, gummy, and resinous components, is produced by honeybees from plant materials [17]. Propolis contains various compounds, such as flavonoids, terpenes, *β*-steroids, aromatic aldehydes, and alcohols [18]. Propolis exhibits a wide range of biological activities, including antibacterial, antifungal, anti-inflammatory, immunostimulatory, antitumor, and antioxidant effects [18]. Therefore, propolis is extensively used in food and beverages to improve health and prevent diseases.

In recent years, considerable efforts have been focused on identifying naturally occurring herbal medicines [19–22]. The flavonoid fraction is the main component of propolis. Many flavonoids exhibit strong antioxidant activity [17]. Our research has focused on studying the cardioprotective properties of the flavonoids extraction from propolis (FP). Our preliminary results showed that FP exerts an effective role in cardiac protection. However, the protective effect of FP on pathological cardiac hypertrophy (PCH) and HF remains unknown. This study shows that FP is a novel cardioprotective agent. First, FP significantly attenuates adverse cardiac dysfunction, hypertrophy, and associated disorder, such as fibrosis. Secondly, FP significantly attenuates apoptotic damage and cardiac remodeling, which is correlated with the PI3K/AKT signaling. Thus, FP may be clinically used to inhibit PCH progression and prevent HF.

## 2. Materials and Methods

### 2.1. Preparation of FP

Propolis was purchased from the An Guo herbal medicine market in Hebei province, China. The propolis was authenticated by Professor Dacheng Jiang from the Changchun University of Chinese Medicine and a voucher specimen (number 1019907) was deposited at the Jilin museum of Materia Medica, Jilin province, China. The sample was frozen at 4°C before being ground into a powder. Then, 10x of 75% ethanol/water was added to extract the active fractions. This process was conducted twice and the fractions were filtered through a 0.45 *μ*m membrane after being combined. The filtrate was evaporated to dryness at 80°C under reduced pressure. One g of the powder was dissolved in 100 mL chloroform-ethanol (10 : 1) and was extracted twice by the same volume of 1% NaOH solution. The aqueous alkali was adjusted to pH 6 by 1% HCl and then left at room temperature for another 24 h for precipitation. The FP were then obtained through filtration, rinsed in water to neutral pH, and dried under reduced pressure.

### 2.2. High Performance Liquid Chromatography-High-Resolution Mass Spectrometry (HPLC-HRMS) Analysis of FP

The FP was dissolved in pure methanol and filtered through a 0.22 *μ*m membrane before HPLC-Q-TOF-MS analysis. The liquid chromatography separation was performed on an Agilent SB-Aq column (250 mm × 4.6 mm, 4.5 *μ*m, 400 bar) at 30°C. For HPLC analysis, 0.1% formic acid/water (v/v) and 0.1% formic acid/methanol were used as the mobile phases A and B, respectively. The gradient elution was programmed as follows: 0–110 min (35–90% B) and 110–120 min (90% B). The flow rate was 1.0 mL/min and the injected sample volume was 10 *μ*L. The Q-TOF-MS scan range was set at *m*/*z* 100–1200 in positive modes. The dry gas (N_2_) flow rate was 9.0 L/min, the dry gas temperature was 350°C, the nebulizer gas was set at 30 psi, the capillary voltage was 3500 V, the fragmentor was 175 V, and the skimmer was 65 V.

Standard pinocembrin, kaempferol, isosakuranetin, pinobanksin-3-O-acetate, 12-acetoxyviscidone, galangin, and chrysin (purity > 98% by HPLC) were purchased from the National Institutes for Food and Drug Control of China. They were mixed and dissolved in pure methanol at the concentration of 2 *μ*M per compound prior to use.

Data analysis was performed on an Agilent MassHunter Workstation Software-Qualitative Analysis (version B.04.00, Build 4.0.479.5, Service Pack 3, Agilent Technologies, Inc., 2011).

### 2.3. Animals and* In Vivo* Pharmacological Treatment

The mice used in this study were handled in compliance with the guidelines for the care and use of laboratory animals established by the Chinese Council on Animal Care, and all animal protocols were approved by the Jilin University Animal Care and Use Committee. Eight-week-old male mice were anesthetized with 1.5% isoflurane. The adult mice were intragastrically given different doses of FP (1–50 mg·kg^−1^·d^−1^) for 7 d. Alzet osmotic minipumps containing PBS or isoproterenol (ISO) were surgically implanted subcutaneously in the interscapular region of the mouse. ISO was calibrated to release the drug at a rate of 25 mg·kg^−1^·d^−1^ for 7 d to experimentally induce heart hypertrophy. The dose-dependent effect of FP on ISO-induced gene reactivation was determined. FP (50 mg·kg^−1^·d^−1^) did not exert an additional benefit to reduce heart hypertrophy; thus, we selected 25 mg·kg^−1^·d^−1^ for the following experiments. In a separate experiment, mice were pretreated with the selective PI3K antagonist wortmannin (WM) (1 mg·kg^−1^) at 1 h before ISO administration. The PI3K inhibitor doses were selected based on the results of previous studies.

### 2.4. Determination of Cardiac Dysfunction through Echocardiography

The animals were euthanized and the hearts were removed for hypertrophic evaluation. The analysis showed no effect on cardiac function. Cardiac function was examined through echocardiography using a Vevo 770 microultrasound system (VisualSonics, Toronto, Ontario, Canada) as described previously [17]. Briefly, an* in vivo* transthoracic echocardiography of the left ventricle was performed using a 30 MHz scan head interfaced with a Vevo 770. An ultrasound beam was placed on the heart and near the papillary muscles. High-resolution two-dimensional electrocardiogram-based kilohertz visualization was achieved. The parameters of cardiac function were digitally measured on the M-mode tracings and then averaged from three to five cardiac cycles.

### 2.5. Histological Analyses

The animals were euthanized and the hearts were removed for hypertrophic evaluation. Serial sections (4 mm) of heart tissues were stained with hematoxylin-eosin [20] or Masson's trichrome and then visualized using a light microscope as previously described.

### 2.6. Transmission Electron Microscopy

The animals were euthanized and the hearts were removed for hypertrophic evaluation. Heart tissue sections were collected and observed by transmission electron microscopy.

### 2.7. Real-Time RT-PCR

Total RNA was extracted using TRIzol (Invitrogen, Carlsbad, CA). Briefly, 2 mg of total RNA was reverse transcribed using the SuperScript first-strand synthesis system (Invitrogen, Carlsbad, CA, USA). cDNA was synthesized from the isolated RNA. Cycle time values were obtained using real-time RT-PCR with the Power SYBR green PCR master mix (Applied Biosystems, Foster City, CA, USA), the iQ5 real-time PCR detection system, and analysis software (Bio-Rad, Hercules, CA, USA) as previously described [23]. Primers were designed using the Applied Biosystems Primer Express Software (version 2.0) (Table 1).

### 2.8. Western Blot Analysis

Heart tissues were lysed on ice with T-PER tissue or cell protein extraction reagent (Pierce Chemical Co., Rockford, IL) containing 0.1 mM dithiothreitol and proteinase inhibitor cocktail. Lysate preparation and Western blot analysis were performed as previously described [21]. Protein concentration was determined using a Bio-Rad DC protein determination kit with BSA as the standard. Immunoblots were developed using an ECL kit.

### 2.9. Caspase-3, Caspase-8, and Caspase-9 Activity Assay

Caspase-3, caspase-8, and caspase-9 activities were measured using a fluorometric assay kit (BioVision, Mountain View, CA, USA) according to the manufacturer's instructions. The samples were subjected to a Fluoroskan Ascent FL fluorometer (Thermo Fisher Scientific, Waltham, MA, USA) with 400 nm excitation and 505 nm emission wavelengths. The results were expressed as fold change compared to the control.

### 2.10. Biochemical Measurements

The protein levels of ANF and *β*-MHC were quantified using ELISA according to the manufacturer's instructions (R&D Systems, Wiesbaden, Germany).

### 2.11. Data Analysis

The data are expressed as the mean ± SE. The significance of the differences between the means was assessed using Student's *t*-test, and *p* values lower than 0.05 were considered significant. One-way ANOVA and Bonferroni corrections were used to determine the significance for multiple comparisons. Calculations were performed using SPSS (version 11.0) statistical software.

### 2.12. Materials

All chemicals were purchased from Sigma (St. Louis, MO) and all antibodies were purchased from Santa Cruz Biotechnology (Santa Cruz, CA).

## 3. Results

### 3.1. Chemical Profiling of FP

**Figure 1:**
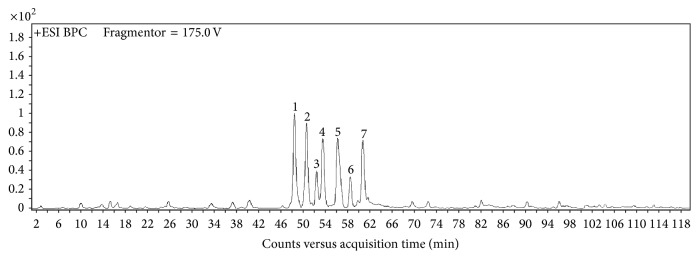
LC-MS base peak chromatograms of FP. The peak numbers refer to Table 1. FP (0–120 min).

By HPLC-Q-TOF-MS analysis, 7 well-separated chromatographic peaks in propolis flavonoids were identified in the BPC of FP (Figure 1). Six of the peaks were characterized as pinocembrin, kaempferol, isosakuranetin, pinobanksin-3-O-acetate, 12-acetoxyviscidone, galangin, and chrysin by comparison to the external standards and the mass data relative abundance and fragmentation rules according to reported references [24–27] (Table 2). And the extraction efficiency of the flavonoids extraction from propolis is 31.32% as determined by colorimetric methods. The purity of flavonoids is 56.13% in chloroform-ethanol solution and increased to 79.88% after extracted by NaOH solution.

### 3.2. FP Dose-Dependently Repressed ISO-Induced Fetal Gene Reactivation

**Figure 2:**
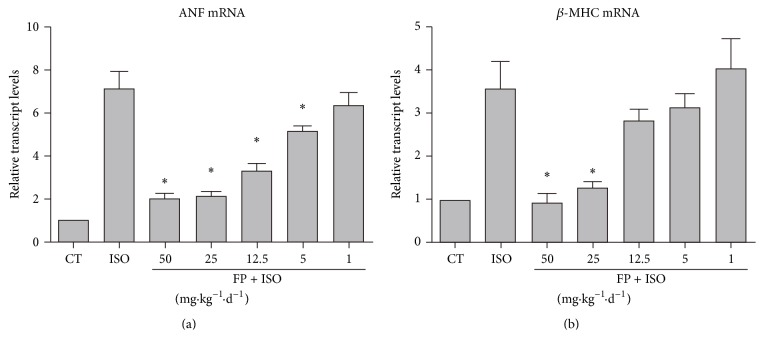
FP represses isoproterenol-induced fetal gene reactivation in a dose-dependent manner. Intragastric injection of various doses of FP (1–50 mg·kg^−1^·d^−1^) for 7 days was followed by continuous infusion with isoproterenol (ISO, 25 mg·kg^−1^·d^−1^) for 7 days to experimentally induce heart hypertrophy. Heart tissues were collected and assayed for ANF (a) and *β*-MHC expression (b). The results are expressed as the means ± SE; *n* = 10 mice per group (^*∗*^ *p* < 0.05 compared with isoproterenol group).

Figure 2(a) shows that intragastric injection of various doses of FP (1–50 mg·kg^−1^·d^−1^) for 7 days was followed by continuous infusion with isoproterenol (ISO, 25 mg·kg^−1^·d^−1^) for 7 days to experimentally induce heart hypertrophy. Heart tissues were collected and assayed to determine ANF and *β*-MHC expression. FP (50 mg·kg^−1^·d^−1^) exerted no additional benefit to reduce heart hypertrophy; thus, we selected 25 mg·kg^−1^·d^−1^ for the following experiments.

### 3.3. FP Attenuated ISO-Induced Cardiac Dysfunction in Mice

To determine whether FP could inhibit cardiac dysfunction* in vivo*, we performed echocardiography in the mice treated with various drugs. The representative echocardiographic parameters are depicted in Table 3. The mice infused with ISO showed an enlarged left ventricular chamber size, indicated by the significant increase in the left ventricular internal dimension values at the end diastole (LVIDd) and end systole (LVIDs) compared to those treated with saline (Table 3). Consistently, these structural alterations were accompanied with marked impairment in cardiac contractile function, represented by the decrease in the left ventricular fractional shortening (FS) and ejection fraction (EF). The treatment with FP significantly reversed the changes stimulated by ISO in these parameters (Table 3).

### 3.4. Reduced Cardiac Hypertrophy in Mice Treated with FP

**Figure 3:**
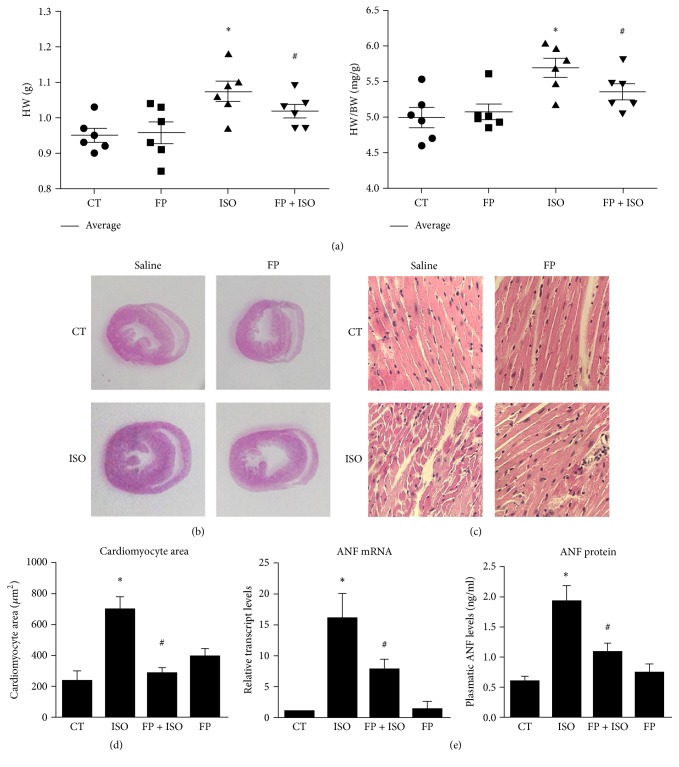
Cardiac hypertrophy is reduced in mice treated by FP. Intragastric injection of FP (25 mg·kg^−1^·d^−1^) for 7 days followed by continuous infusion with isoproterenol (ISO, 25 mg·kg^−1^·d^−1^) for 7 days was sued to experimentally induce heart hypertrophy. (a) Animals were euthanized and the hearts were removed for hypertrophic evaluation. A comparison of HW (g) and HW/BW ratios (mg/g) for the animals is shown. (b) Representative histological sections of hearts stained with H&E. (c) Magnified images of histological sections in (b) used to determine CM cross-sectional area. (d) Quantification of CM cross-sectional area in the left ventricular wall. (e) mRNA expression and plasma protein levels of the hypertrophy marker ANF. The results are expressed as the means ± SE; *n* = 10 mice per group (^*∗*^ *p* < 0.05 compared to the control group; ^#^ *p* < 0.05 compared to the isoproterenol group).

To further verify the antihypertrophic effect of FP, we directly measured the heart weight (HW) and heart-to-body weight (HW/BW) ratio after the animals were euthanized. As shown in Figure 3(a), after 7 d of ISO infusion, HW significantly increased in ISO-treated mice compared to saline-treated mice. However, FP treatment attenuated the increased HW and HW/BW ratio.

Histological sections were also analyzed using hematoxylin-eosin staining (Figures 3(b)–3(d)), whereas ANF mRNA and protein levels were quantified through qPCR (Figure 3(e)) and ELISA assay (Figure 3(e)). As shown in Figure 3(d), the CM cross-sectional area was larger in ISO-treated mice than in saline-treated mice. FP treatment attenuated the increased cross-sectional area in ISO-treated mice. The expression of the hypertrophic marker ANF also increased in ISO-treated mice compared to saline-treated mice. Moreover, FP treatment reduced the increased mRNA and protein levels of ANF. However, FP alone had no significant effect on these processes.

### 3.5. FP Treatment Reduced Associated Disorder, Such as Fibrosis

**Figure 4:**
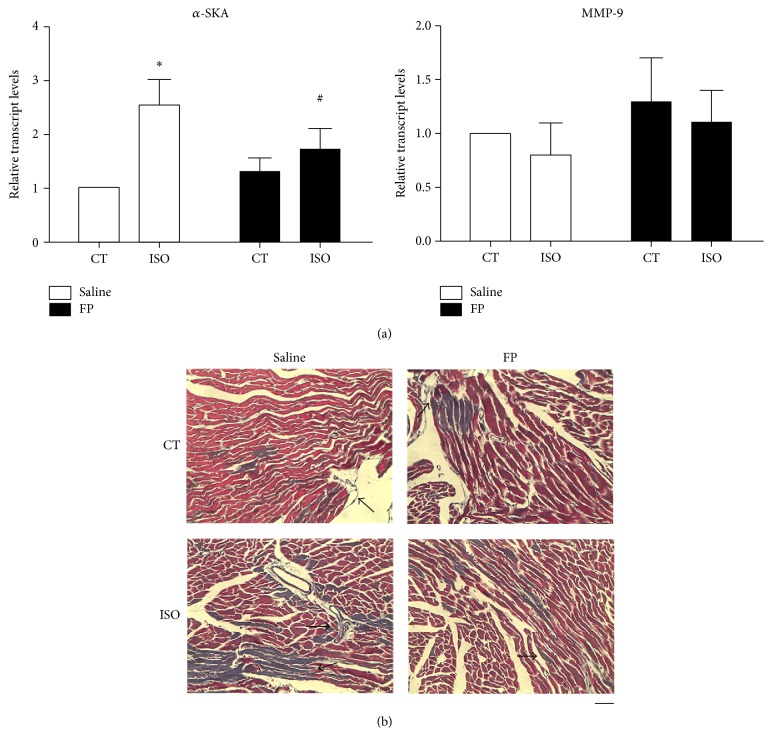
FP treatment reduced associated disorder, such as fibrosis. (a) mRNA expression levels of the hypertrophy marker a-SKA and the fibrosis marker matrix metalloproteinase-9 (MMP-9). (b) Determination of fibrosis in histological sections by Masson's trichrome staining. Scale bar, 50 *μ*m. The arrows show fibrotic areas. The results are expressed as the means ± SE; *n* = 10 mice per group (^*∗*^ *p* < 0.05 compared with control group; ^#^ *p* < 0.05 compared with isoproterenol group).

To evaluate fibrosis, we evaluated the expression of *α*-skeletal actin (*α*-SKA) mRNA and matrix metalloproteinase-9 by using Masson's trichrome staining (Figure 4). Consistently, the ISO-treated hearts presented massive cell death with replacement fibrosis (Figure 4(b)). In contrast, FP treatment abbreviated ISO-elicited cardiac cell death/fibrosis. Using both measures, ISO substantially increased the mRNA levels of *α*-SKA (Figure 4(a)). Moreover, FP treatment attenuated the ISO-induced *α*-SKA levels. However, ISO and FP did not significantly affect the MMP-9 levels. Collectively, FP treatment attenuated cardiac hypertrophy and the associated disorder, such as fibrosis.

### 3.6. Effects of ISO and FP on Apoptotic Damage and Apoptosis-Related Gene Expression in Heart Tissues

**Figure 5:**
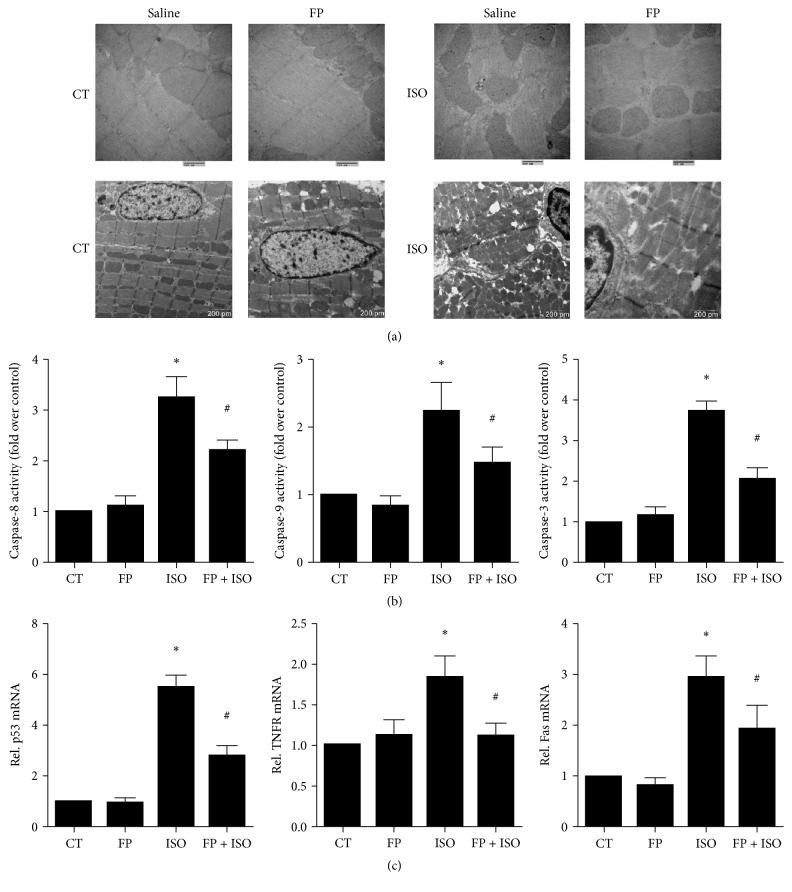
Effects of isoproterenol and FP on apoptotic damage and apoptosis-related gene expression in heart tissues. (a) Transmission electron microscopy of heart tissues. (b) The activities of caspase-8, caspase-9, and caspase-3 were measured using a fluorometric assay and expressed as the fold change over the control. (c) mRNA levels of p53, TNFR1, and Fas were determined by real-time RT-PCR. The levels of mRNA were normalized to GAPDH. Relative mRNA levels are shown using arbitrary units, and the value of the control group (CT) is defined as 1. The results are expressed as the means ± SE; *n* = 10 mice per group (^*∗*^ *p* < 0.05 compared to the control group; ^#^ *p* < 0.05 compared to the isoproterenol group).

Apoptotic damage has been implicated in cardiac hypertrophy. To determine the relationship between the cardioprotection of FP against ISO-induced cardiac hypertrophy and apoptosis, we assessed the morphology of mouse heart tissues using transmission electron microscopy (Figure 5(a)). The results showed evident heart tissue abnormalities, including cytoplasmic vacuolization, myofibrillar loss, mitochondrial edema, chromatin condensation, and cardiomyocyte necrosis, in the cardiac sections of ISO-induced mice. The structural abnormalities in the hearts were partially prevented by cotreatment with FP (Figure 5(a)). Moreover, the activation of caspase-3 resulted in the cleavage of PARP. Caspase-3 activation is one of the key processes involved in apoptosis and contributes to myocardial dysfunction and cardiac hypertrophy.  Figure 5(b) shows that myocardial caspase-3 activation was reduced in the mice cotreated with FP and ISO compared to the mice treated with ISO alone, as indicated by the increased activity.  Figure 5(c) shows that apoptotic damage was activated in the myocardium of ISO-infused mice. This finding was depicted by the elevated caspase-8, caspase-9, and caspase-3 activities (Figure 5(b)) and the increased mRNA levels in p53, TNF-R1, and Fas, which were attenuated by FP treatment (Figure 5(c)).

### 3.7. FP Prevented ISO-Induced Cardiac Remodeling* In Vivo*, Which Is Correlated with the PI3K/AKT Signaling

**Figure 6:**
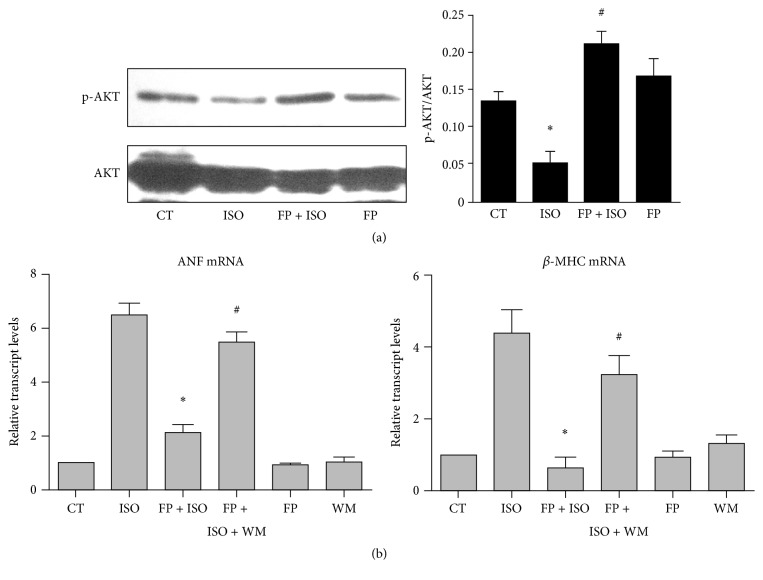
FP prevents isoproterenol-induced cardiac remodeling* in vivo*, which is correlated with PI3K/AKT signaling. (a-b) Intragastric injection of FP (25 mg·kg^−1^·d^−1^) or saline (CT) for 7 days was followed by continuous infusion with isoproterenol (ISO, 25 mg·kg^−1^·d^−1^) for 7 days to experimentally induce heart hypertrophy. The animals were euthanized and the hearts were removed for hypertrophic evaluation. (a) Representative Western blotting assays and quantitative analysis of phosphorylated AKT in the hearts by drug treatment (^*∗*^ *p* < 0.05 compared to the control group; ^#^ *p* < 0.05 compared to the isoproterenol group). (b) Effect of a selective PI3K antagonist, wortmannin (WM), on isoproterenol-induced gene reactivation. The heart tissues were collected and assayed for ANF and *β*-MHC expression. The results are expressed as the means ± SE; *n* = 10 mice per group (^*∗*^ *p* < 0.05 compared to the isoproterenol group; ^#^ *p* < 0.05 compared to the FP-isoproterenol cotreated group).

As shown in Figure 6(a), Western blot analysis revealed significantly enhanced AKT phosphorylation in the FP-treated hearts even if the total AKT protein was similar in all animal groups. FP-ISO cotreated mice exhibited higher p-AKT in the hearts than those treated with FP alone; however, the difference was not significant. These results indicated that the cardioprotective effect of FP could be attributed to the antihypertrophic and antiapoptotic activities, which possibly involved the PI3K-AKT signaling pathway.

We also determined the functional consequences of disrupting the PI3K-AKT signaling pathway in pathological hypertrophy induction. WM, a selective PI3K inhibitor, was used to suppress PI3Ks. The results were consistent with the findings shown in Figure 2. ISO immediately upregulated the expression of ANF and *β*-MHC (Figure 6(b)), whereas FP treatment markedly antagonized ISO-induced fetal gene expression (Figure 6(b)). However, the ISO-induced increase in the ANF and *β*-MHC mRNAs was attenuated by FP in the mice pretreated with WM (Figure 6(b)), whereas FP and WM showed no significant effect compared to the control. Furthermore, as is shown in supplementary figure (see Supplementary Material available online at http://dx.doi.org/10.1155/2016/6281376), cardiac hypertrophy phenotype is not altered by WM. These results suggested that the inhibition of PI3Ks with WM could suppress the FP-induced antihypertrophic effect.

## 4. Discussion

Natural drugs that exert cardioprotective properties have gained worldwide attention [9, 13]. Propolis, a traditional medicine, has been widely used for a thousand years as an anti-inflammatory and antioxidant drug. However, the role of propolis in cardiovascular diseases remains unknown. Propolis possesses a wide range of biological activities. Total flavonoids are the main active component of propolis and exhibit strong antioxidant activities. Therefore, this study assessed the cardioprotective effects of FP in the ISO-induced C57BL/6 mouse model, as well as the mechanisms that mediate the therapeutic activities of this drug. The significant findings of this study were as follows: (1) FP treatment significantly attenuated adverse cardiac dysfunction, hypertrophy, and associated disorder, such as fibrosis; (2) FP treatment significantly attenuated apoptotic damage and cardiac remodeling, which were correlated with the PI3K/AKT signaling.

Cardiac hypertrophy can be divided into two categories, physiological hypertrophy and pathological hypertrophy. It is generally recognized that ANP level is increased in hypertrophic hearts. However, some results also supported that factors can protect against pathological cardiac hypertrophy by activating PI3K/AKT signaling [28]. In accordance with our study, factors can also attenuate pathological cardiac hypertrophy by increasing AKT, and effects can be blocked with PI3K inhibition [23, 29, 30]. Furthermore, some factors such as Cav-3 protect against cardiac hypertrophy and ischemia by increasing p-AKT signaling and ANP, partially due to mimicking ischemia-induced preconditioning [31, 32]. These seemingly opposite results may be partially ascribed to multiple functions of PI3K and complicated experimental conditions varied in the settings of cardiac hypertrophy. Although PI3K is a well-known survival factor which can inhibit cardiac apoptosis and cell death, in some conditions such as exercise it can activate PI3K/AKT signaling and leads to cardiac hypertrophy, with little effect on cell death. Previous experiments have shown that insulin or IGF-I may regulate heart development [33]. PI3K of the IA group could be activated by IGF-I through the binding of ligands to PI3K*α* [34, 35]. After activation of PI3K, plasma membrane lipid phosphatidylinositol-4,5-bisphosphate is converted to phosphatidylinositol-3,4,5-triphosphate, which may lead to the activation of a series of signaling components. To the best of our knowledge, our present study indicates that FP preserves cardiac function with a decrease in levels of ANF and *β*-MHC, and effects can be inhibited by PI3K antagonist, wortmannin. And FP induces activation in PI3K/AKT signaling and induction in antiapoptotic and antioxidative properties may also have complex effects on cardiac hypertrophy signaling.

Pathological cardiac hypertrophy (PCH) is a vital and independent predictor of cardiovascular mortality and is also a key target in the treatment of HF [36–39]. Studies have shown that ISO-induced HF is associated with an array of metabolic and biomedical disorders in experimental animals [2, 23]. ISO infusion causes myocardial ischemia and cardiomyocyte necrosis and progressively leads to diastolic and systolic dysfunction in the heart, which eventually results in PCH and HF [3]. In the present study, we also observed a significant cardiac dysfunction caused by ISO infusion, as indicated by the enlarged left ventricular chamber size and marked impairment in cardiac contractile function, which can be partially attenuated by FP. This finding was closely correlated with our data that FP can attenuate PCH and fibrosis marker expression. PCH is characterized by increased cell volume and metabolic and biochemical disorders, accompanied by reactivation of fetal cardiac genes such as ANF and *β*-MHC. In this study, we first investigated the effects of FP on the mRNA expression of these two fetal cardiac genes. PCH is a maladaptive response that could result in HF when unmatched cardiac cell death and fibrosis occur. Collectively, our study revealed that FP treatment attenuated cardiac hypertrophy and associated disorder, such as fibrosis.

Apoptotic damage has been implicated in cardiac hypertrophy [40, 41]. Apoptosis of cardiac cells is caused by biomedical stimuli that eventually lead to morphological changes in these cells. The signals involved in cardiac hypertrophy may finally result in cell death. Because cardiomyocytes are nondividing cells, the apoptotic cardiomyocyte may be largely replaced by fibrous tissues [42]. Hence, cardiac cell death has a close association with the transition of hypertrophy to irreversible HF [41, 42]. Our present study clearly showed that FP attenuated the apoptotic damage activated in the myocardium of ISO-induced mice, as evidenced by attenuating structural abnormities and decreasing the elevated caspase-8, caspase-9, and caspase-3 activities and the increased mRNA levels of p53, TNF-R1, and Fas. These results revealed that the recovery of PCH and cardiac dysfunction induced by ISO infusion may be closely associated with diminished cardiac cell apoptosis after FP pretreatment. To the best of our knowledge, this study is the first to demonstrate the beneficial effects of FP on ISO-induced PCH in mice.

## 5. Conclusions

In summary, this study demonstrates that FP has potent cardioprotective activities against ISO-induced HF in the C57BL/6 mouse, and these activities are correlated with PI3K/AKT signaling. Additional experiments should be performed to investigate the cardiac protection properties of FP. FP may be a novel therapeutic agent for inhibiting the progression of PCH and HF.

## Supplementary Material

A selective PI3K antagonist, wortmannin (WM), on HW (g), HW/BW ratios (mg/g) for the animals and quantification of CM cross-sectional area in the left ventricular wall are shown.

## Figures and Tables

**Table 1:** Primers used for real-time RT-PCRs.

Target genes	Primer pairs (5′-3′)	
	Forward	Reverse
*ANF*	AGCATGGGCTCCTTCTCCAT	TGGCCTGGAAGCCAAAAG
*β-MHC*	CCTACAAGTGGCTGCCTGTGT	ATGGACTGATTCTCCCGATCTG
*α-SKA*	GGAGAAGATCTGGCACCATACATT	AGCAGGGTTGGGTGTTCCT
*MMP-9*	GGACGACGTGGGCTACGT	CACGGTTGAAGCAAAGAAGGA
*p53*	CAAAAGAAAAAACCACTTGATGGA	CGGAACATCTCGAAGCGTTTA
*TNFR*	CTCAGGTACTGCGGTGCTGTT	GCACATTAAACTGATGAAGATAAAGGA
*Fas*	GCTGCGCCTCGTGTGAA	GCGATTTCTGGGACTTTGTTTC
*GAPDH*	AACGACCCCTTCATTGAC	TCCACGACATACTCAGCAC

**Table 2:** Characteristics of chemical components of TFP by HPLC-HRMS.

Peak number	Retention time (min)	*m*/*z* for [M + H]^+^	MS^*n*^ [M + H]^+^	Formula	Identification	Relative abundance (%)
1	48.499	257.0824	215, 153, 131	C_15_H_12_O_4_	Pinocembrin	6.74
2	50.621	287.0950	165, 153, 121	C_15_H_10_O_6_	Kaempferol	6.01
3	52.497	287.0931	245, 217	C_16_H_14_O_5_	Isosakuranetin	1.71
4	53.480	315.0867	273, 255	C_17_H_14_O_6_	Pinobanksin-3-O-acetate	4.1
5	56.231	277.0852	232, 216	C_15_H_16_O_5_	12-Acetoxyviscidone	6.51
6	58.527	271.0615	165, 153, 105	C_15_H_10_O_5_	Galangin	1.77
7	60.755	255.0676	209, 153, 129	C_15_H_10_O_4_	Chrysin	4.47

**Table 3:** Echocardiography data after ISO-induced hypertrophy.

	Saline		TFP	
	CT (*n* = 5)	ISO (*n* = 5)	CT (*n* = 5)	ISO (*n* = 5)
LVIDs (mm)	2.09 ± 0.31	3.01 ± 0.28^*∗*^	2.11 ± 0.21	2.25 ± 0.35^#^
LVIDd (mm)	3.60 ± 0.56	4.34 ± 0.34^*∗*^	3.72 ± 0.42	3.95 ± 0.51^#^
LVVs (*μ*L)	14.29 ± 3.17	35.3 ± 8.04^*∗*^	18.35 ± 4.20	17.26 ± 5.12^#^
LVVd (*μ*L)	54.28 ± 10.63	84.74 ± 19.28	63.89 ± 13.11	76.44 ± 14.51
EF (%)	75.23 ± 8.34	58.34 ± 5.91^*∗*^	71.28 ± 11.55	77.42 ± 10.01^#^
FS (%)	43.20 ± 6.03	30.58 ± 4.67^*∗*^	39.94 ± 6.98	45.76 ± 9.20^#^
SV (*μ*L)	39.99 ± 11.56	49.44 ± 12.64	45.55 ± 13.11	59.18 ± 10.03

LVIDs, left ventricular internal diameter at systolic phase; LVIDd, left ventricular internal diameter at diastolic phase; LVVs, left ventricle end-systolic volume; LVVd, left ventricle end-diastolic volume; EF, ejection fraction; FS, fractional shortening; SV, stroke volume. All measurements are means ± SE. The data were analyzed by one-way ANOVA (^*∗*^ *p*< 0.05 compared to saline controls; ^#^ *p*< 0.05 compared to ISO-induced saline-treated animals).
