# Supplementary material for: Flavonoids Extraction from Propolis Attenuates Pathological Cardiac Hypertrophy through PI3K/AKT Signaling Pathway
Source: Evid Based Complement Alternat Med. 2016 Apr 24;2016:6281376. doi: 10.1155/2016/6281376 (PMC4860246; doi:10.1155/2016/6281376)
Supplement: Supplementary file 1 — A selective PI3K antagonist, wortmannin (WM), on HW (g), HW/BW ratios (mg/g) for the animals and quantification of CM cross-sectional area in the left ventricular wall are shown. [file 6281376.f1.pdf]

Suplementary figure

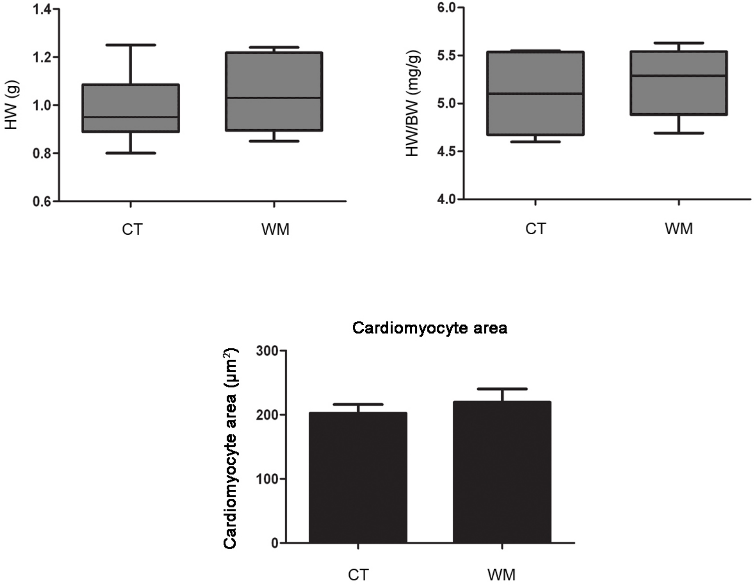

Supplementary figure Cardiac hypertrophy phenotype is not altered by WM. A selective PI3K antagonist, wortmannin (WM), on HW (g), HW/BW ratios (mg/g) for the animals and quantification of CM cross-sectional area in the left ventricular wall are shown. The results are expressed as the means ± SE, n=5 mice per group (*$p < 0.05$ vs. CT).
